# Coincidence of Intra-Abdominal Splenosis in a Patient with Advanced Ovarian Cancer: Case Report and Review of the Literature

**DOI:** 10.1055/s-0041-1731426

**Published:** 2021-06-23

**Authors:** Tatjana Braun, Amelie De Gregorio, Lisa Baumann, Jochen Steinacker, Wolfgang Janni, Nikolaus De Gregorio

**Affiliations:** 1 Department of Gynecology and Obstetrics, University Hospital Ulm, Ulm, Germany; 2 Department of Pathology, University Hospital Ulm, Ulm, Germany; 3 Department of Diagnostic and Interventional Radiology, University Hospital Ulm, Ulm, Germany

**Keywords:** diagnostic imaging, gynecologic oncology, gynecologic operation, ovarian neoplasms, splenosis

## Abstract

Splenosis is a rare disease, which is often discovered incidentally years after surgical procedures on the spleen or traumatic splenic lesions. Through injury of the splenic capsule, splenic cells are able to spread and autoimplant in a fashion similar to the process of metastatic cancer. Here we present the case of a 62-year-old female patient with a palpable tumor of the lower abdomen. Her medical history was unremarkable, except for splenectomy after traumatic splenic lesion in her childhood. Clinical examination and diagnostic imaging raised the suspicion of advanced ovarian cancer, which was further substantiated by the typical presentation of adnexal masses and disseminated peritoneal metastases during the following staging laparotomy. Surprisingly, we also found peritoneal implants macroscopically similar to splenic tissue. Microscopic examination of tissue specimens by intrasurgical frozen section confirmed the diagnosis of intra-abdominal splenosis. The patient then underwent cytoreductive surgery with complete resection of all cancer manifestations, sparing the remaining foci of splenosis to avoid further morbidity. This case demonstrates the rare coincidence of intra-abdominal carcinoma and splenosis, which could lead to intraoperative difficulties by misinterpreting benign splenic tissue. Therefore, splenosis should be considered in patients with medical history of splenic lesions and further diagnostic imaging like Tc-99m-tagged heat-damaged RBC scan could be used for presurgical distinguishing between tumor spread in the abdominal cavity and disseminated splenosis. The presented case report should not only raise awareness for the rare disease splenosis, but also emphasize the need to consider the possibility of simultaneous incidence of benign and malignant intra-abdominal lesions, as to our knowledge this is the first published case of simultaneous peritoneal carcinomatosis and splenosis.

Splenosis is a rare disease, which is often discovered incidentally years after surgical procedures on the spleen or traumatic splenic lesions with subsequent splenectomy. Through injury of the splenic capsule, splenic cells are able to spread into the abdominal cavity comparable to disseminated peritoneal metastases. In the majority of cases with reported splenosis, the splenic tissue organizes itself like a congenital accessory spleen on various ectopic intra-abdominal locations. There are rare cases, especially with additional laceration of the diaphragm, where splenic tissue is even found in the thoracic cavity. 1 2

## Case Report and Case Presentation

**Fig. 1:**
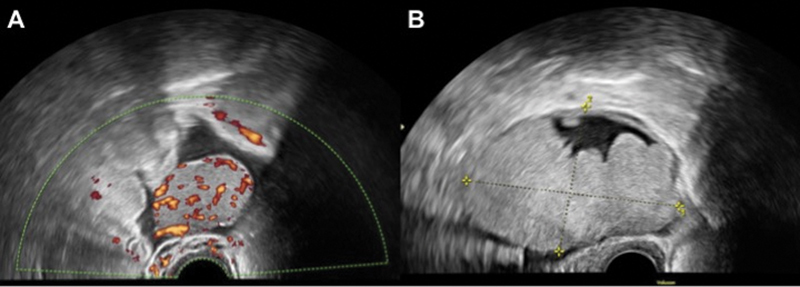
( **A** ) Ultrasound image of the right ovary: 38 × 28 mm large, homogeneous, solid and hypervascularized tumor. ( **B** ) Ultrasound image of the left ovary: 80 × 54 mm large, homogeneous, solid-cystic and hypervascularized tumor.

A 62-year-old patient (II Gravida/II Para) presented with diarrhea for several weeks and a palpable tumor of the lower abdomen. Her medical history was unremarkable, except for having had a splenectomy after traumatic splenic lesion in her childhood. Clinical examination showed a palpable mass in the lower abdomen without any further anomalies. Using vaginal ultrasound, we detected ascites and suspicious adnexal masses. On the right ovary, there was a 38 × 28 mm large, homogeneous, solid, hypervascular tumor ( Fig. 1A ) and on the left ovary, we found an 80 × 54 mm large, solid-cystic, hypervascular structure ( Fig. 1B ). The laboratory workup revealed increased CA 125 levels (403 U/mL, elevated 11.5× upper norm limit).

**Fig. 2:**
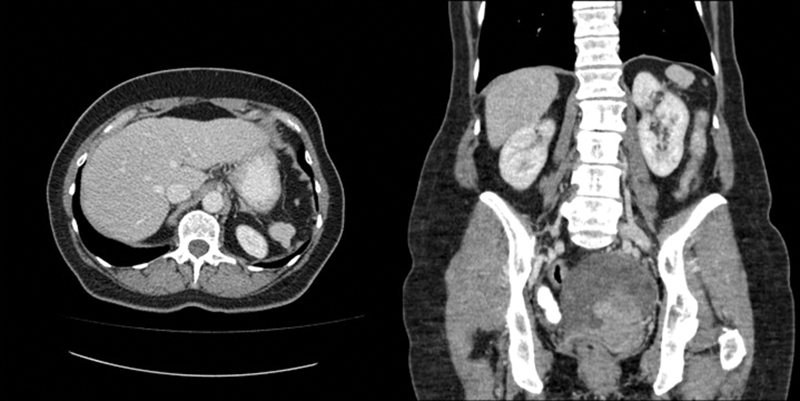
Presurgical portal venous phase computer tomography scan with more than 10 smoothly bordered, homogeneous, contrast enhancing lesions, most of them in the peripancreatic fatty tissue as well as adjacent to the greater curvature of the stomach, perihepatic and mesenterial. Several of the tumors are in the left subphrenic space. One of the tumors was located at the right pelvic wall with contact to the colon and rectum as well as to a big inhomogeneous retro-uterine tumor in the small pelvis, without separating tissue. The perihepatic, peripancreatic an mesenterial lesions were isodense to hyperdense compared with the liver parenchyma (density of 104 to 122 Hounsfield units).

Further diagnostic workup included a coloscopy and gastroscopy without any pathological findings and a computer tomography (CT) scan of the abdomen and thorax ( Fig. 2 ). In the portal venous phase of the CT scan, we detected more than 10 smoothly bordered, homogeneous, and contrast-enhancing lesions, primarily in the peripancreatic fatty tissue and in addition adjacent to the greater curvature of the stomach, perihepatic, mesenterial, and several tumors in the left subphrenic space. One of the lesions was located at the right pelvic wall with contact to the colon and rectum as well as to a large inhomogeneous retro-uterine tumor in the small pelvis without separating tissue. The perihepatic, peripancreatic, and mesenterial lesions were isodense to hyperdense compared with the liver parenchyma (density of 104 to 122 Hounsfield units [HU]). Based on the preoperative suspicion of advanced ovarian cancer (FIGO IIIc), the patient underwent staging laparotomy for cytoreductive surgery.

**Fig. 3:**
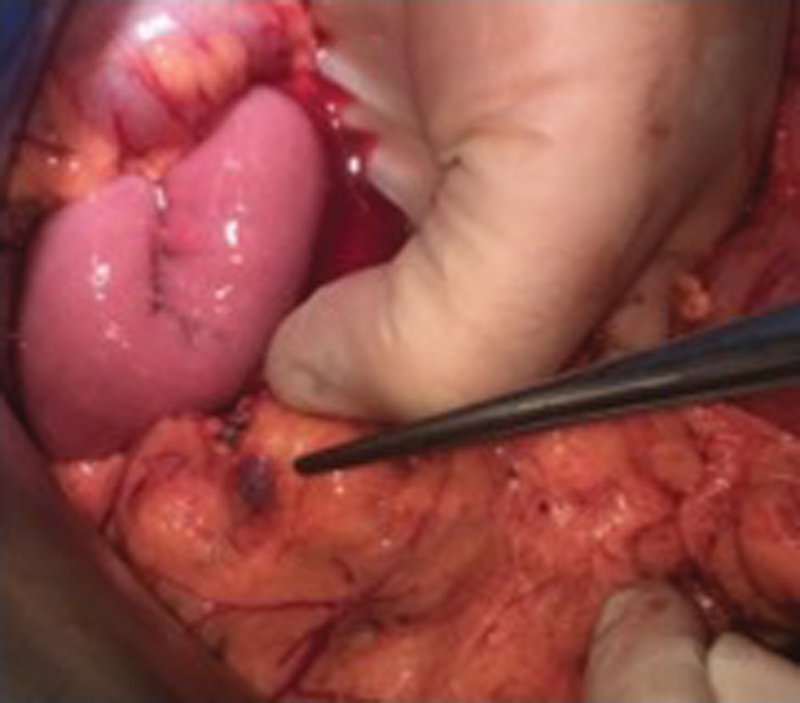
Intraoperative presentation of splenosis in the greater omentum.

**Fig. 4:**
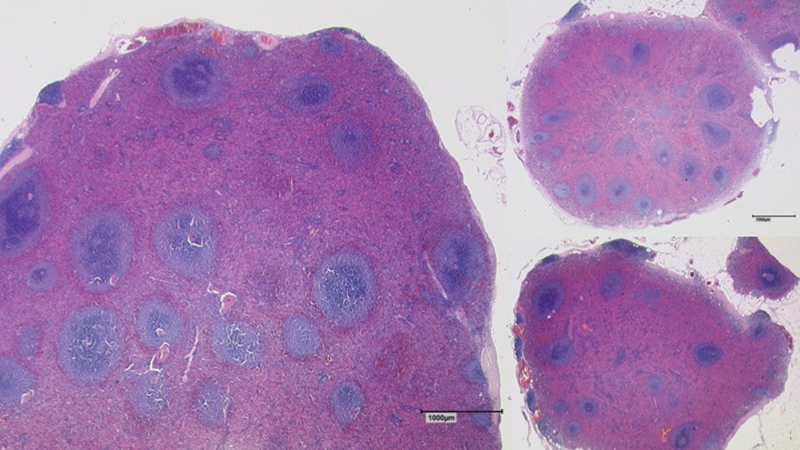
Histological presentation of splenosis. The tissue shows the typical structure of splenic tissue with red and white pulp covered by a fibrous capsule from which trabeculae enter into the parenchyma (Hematoxylin and Eosin staining, magnification ×25, measuring bar 1,000 µm).

The intraoperative situs showed the typical appearance of advanced ovarian cancer with disseminated peritoneal metastases. In addition to superficial tumor spread in the abdominal cavity and adnexal masses, there were multiple peritoneal implants, involving the mesentery and serosa of the small intestine, macroscopically similar to splenic tissue ( Fig. 3 ). Although the lesions showed distinct morphological features, distinguishing them from peritoneal carcinomatosis, one of the largest nodes (40 × 25 × 20 mm) covering the rectum was resected and send to intrasurgical frozen section assessment, to histologically confirm that the nodules were not another manifestation of the advanced ovarian cancer. The lesion was then identified as intra-abdominal splenosis. Final histopathological analysis corroborated the diagnosis. The resected tissue showed the typical structure of splenic tissue with red and white pulp covered by a fibrous capsule from which trabeculae entered into the parenchyma ( Fig. 4 ). Histopathological examination of the resected greater omentum yielded 12 additional foci of splenosis.

With the intrasurgical confirmation of splenosis, the remaining foci of splenosis could be spared and surgery was limited to clinically suspected cancer manifestations. Cytoreductive surgery comprised hysterectomy, bilateral adnexectomy, deperitonealization of the recto-uterine pouch, omentectomy, appendectomy, and resection of several peritoneal cancer lesions from the mesentery and resulted in macroscopic complete resection. Histology showed a high grade serous ovarian cancer, pT3b, N0, M0, L0, V0, Pn0 (FIGO IIIb). The patient was hospitalized for 12 days and afterwards underwent chemotherapy with six cycles of carboplatin/paclitaxel, as well as a maintenance therapy with bevacizumab for 1 year. After surgery and the first cycle of chemotherapy, CA125 levels returned to normal (10.5 U/mL) and did not increase again during therapy. We could discharge the patient into oncological follow-up care without any clinical evidence of remaining or recurrent cancer (follow-up time so far 17 months after surgery).

## Discussion and Conclusion

One of the most important goals in treatment of advanced epithelial ovarian cancer— determining overall survival and progression free survival—is complete resection of the tumor mass in primary surgery. 3 However, avoiding unnecessary procedures and limiting surgery strictly to cancer manifestations can reduce the rate of complications and consequently improve patient outcome. Therefore, differentiating between benign lesions and cancer manifestations is of great importance when both of them occur simultaneously. In this particular presented case, the challenge comprised of the differentiation between intra-abdominal manifestations of splenosis and peritoneal cancer. If splenosis had been diagnosed prior to surgery, even the resection of the singular rectal node for histological confirmation could have been avoided. Although our patient did not suffer from complications, operating on the intestine is associated with the risk of intestinal dysfunction and infection of the abdominal cavity and stenosis. Moreover, patients might even benefit from the residual splenotic tissue as there are indications that patients with splenosis might restore at least partial reticuloendothelial function after splenectomy. 4

Despite its rare occurrence, there are other cases described in literature, where splenosis led to complications during surgery. Nicolas et al for example presented a case of a 30-year-old patient, who showed excessive weight gain after gastric band surgery and was therefore indicated for conversion into a gastric sleeve. 5 During the operation, they detected many foci of splenosis, for example, at the gall bladder, the stomach, the liver, and the diaphragm. 5 Because of the high risk of massive bleeding through erosion of the gastric band into the splenic tissue, the gastric band had to be removed and the surgery terminated due to the difficult anatomic situation. 5

Another pitfall was described by Sorensen et al, which shows the consequences of misdiagnosis for the individual patient and strongly emphasizes the importance of awareness. 6 In their case, curative surgery for a patient with carcinoma of the Papilla vateri was denied because of peritoneal carcinosis diagnosed in a CT scan. 6 Although this is a highly uncommon procedure, a biopsy of one of the peritoneal lesions was performed to secure the diagnosis of peritoneal cancer. 6 Histopathological analysis of the biopsy revealed splenic tissue. Thus, the patient could undergo the surgical resection of the tumor via curative Whipple procedure. 6

These cases emphasize the importance of diagnosing splenosis prior to surgery. However, it is a big challenge in clinical routine and especially awareness of this disease is limited. Although the lesions are visible in CT scans with similarity to splenic tissue in density and enhancement of contrast agent, reliable diagnosis is difficult. 7 Magnetic resonance imaging cannot yield further information either in most of the cases the lesions are, similar to the spleen, hypointense on T1-weighted images and hyperintense on T2-weighted images. Furthermore, the manifestations present an irregular enhancement of contrast agent in the arterial phase and a homogeneous enhancement in the late contrast phase. 7 8 9

After imaging diagnostics, several patients were misdiagnosed with malignomas like hepatocellular carcinoma, 10 lung cancer, 2 or gastrointestinal stromal tumor 11 resulting in unnecessary surgical procedures and avoidable morbidity. Therefore, further diagnostic tools are warranted to differentiate splenosis from carcinomas in imaging.

One method is the Tc-99m-tagged heat-damaged RBC scan (Tc-99m-DRCB), where heat denaturated red blood cells are tagged with Tc-99m. These denaturated cells accumulate in the red pulp and can be used to detect splenic tissue. 7 9 12 13 14

Another technique is using scintigraphy with ^111^ Indium-tagged thrombocytes, which gather in the reticuloendothelial system of splenic tissue. 7 13 In rare cases, diagnosis is even further complicated by simultaneous incidence of splenosis and malignant disease, similar to the presented case. Misinterpretation could have fatal consequences for patients as potential curative treatment is replaced by palliative therapy for advanced disease. Preoperative awareness with subsequent additional diagnostics is important to avoid under- or overtreatment in those patients.

Since there are no distinct symptoms of splenosis, patients who underwent splenectomy should generously undergo prior examination for remaining splenic tissue. Especially in the absence of Howell-Jolly-Bodies, the lack of increase in reticulocyte count and the presence of antipneumococcal antibodies in nonvaccinated patients 7 ; one of the earlier mentioned imaging techniques should be considered prior to an exploratory surgery. As we were preoperatively not aware of a potential simultaneous splenosis and the diagnosis was only suspected during surgery, no Tc-99m-DRCB or scintigraphy with ^111^ Indium-tagged thrombocytes was performed in the presented case.

To our knowledge, this is the first published case of simultaneous incidence of peritoneal carcinosis and splenosis. Most of the cases in the literature only report splenosis as a differential diagnosis to peritoneal carcinomatosis. 6 15 16

In conclusion, intra-abdominal splenosis is a rare event in patients who underwent surgical procedures of the spleen or traumatic splenic lesions. Coincidence of splenosis with malignancies can mimic peritoneal cancer spread to the abdominal cavity and may lead to misinterpretation of the tumor extent. However, reliable discrimination of cancer masses and splenosis is essential for the prevention of unnecessary surgery and avoiding further morbidity, as well as for the correct classification of clinical tumor stage and the appropriate treatment scheduling. Consideration of splenosis as a potential differential diagnosis for intra-abdominal tumor lesions is important, especially in patients with corresponding medical history, and physicians should be aware of this rare event also in cases of simultaneous malignancies.
